# Herpes Zoster after COVID-19 Infection or Vaccination: A Prospective Cohort Study in a Tertiary Dermatology Clinic

**DOI:** 10.1155/2023/2206498

**Published:** 2023-12-31

**Authors:** Charussri Leeyaphan, Pattriya Jirawattanadon, Sumanas Bunyaratavej, Waratchaya Panjapakkul, Thrit Hutachoke, Yanisorn Nanchaipruek, Phumithep Phumariyapong

**Affiliations:** Department of Dermatology, Faculty of Medicine Siriraj Hospital, Mahidol University, Bangkok, Thailand

## Abstract

**Background:**

Herpes zoster (HZ) has been observed to occur after COVID-19 infection and vaccination; however, knowledge regarding the demographic data, clinical presentations, and treatment outcomes of HZ is limited.

**Objective:**

To compare the demographic data, clinical manifestations, treatments, and outcomes of patients with and without HZ within 14 days of COVID-19 infection or vaccination.

**Methods:**

This prospective cohort study involving patients diagnosed with cutaneous HZ was conducted at a dermatology clinic from October 2021 to January 2023.

**Results:**

Among a total of 232 patients with HZ, the median age was 62.0 years and 59.1% were female. HZ developed in 23 (9.9%) and four (1.7%) patients after COVID-19 vaccination and infection, respectively. The mean duration from vaccination and the median duration from infection to HZ onset were 5.7 and 8.5 days, respectively. The proportion of female patients was significantly higher in the group of patients with COVID-19 vaccination or infection than in those without such a history (*P* = 0.035). Patients who developed HZ following the recent COVID-19 infection had a median age of 42.5 years, which was lower than that of the other groups. Dissemination occurred in 8.7% of the patients after COVID-19 vaccination. HZ recurrence was reported in five cases, of which 80% had been vaccinated or infected with COVID-19 during the previous 21 days. All patients had similar durations of antiviral treatment, crust-off time, and duration of neuralgia.

**Conclusions:**

HZ after COVID-19 vaccination is more frequently observed in females, while HZ after COVID-19 infection tends to occur in younger patients. Disseminated HZ is more common in patients recently vaccinated against COVID-19. COVID-19 vaccination or infection may trigger recurrent HZ infection.

## 1. Introduction

Herpes zoster (HZ) is the reactivation of the varicella-zoster virus (VZV) in the sensory ganglia after a long latency period of the primary varicella infection. It presents as a group of painful, erythematous, maculopapular, or vesicular rashes with a unilateral dermatomal distribution [1, 2]. The prevalence of HZ increases with advancing age [3, 4] and is more common among individuals with an immunocompromised status [5] due to a decline in cell-mediated immunity (CMI) [1, 6, 7]. Furthermore, there have been reports of an increase in the prevalence of HZ during the coronavirus disease (COVID-19) pandemic [8, 9].

COVID-19 is caused by severe acute respiratory syndrome coronavirus 2 (SARS-CoV-2) and is transmitted through respiratory droplets, direct contact, and the fecal-oral route [10–12]. The clinical manifestations of COVID-19 range from asymptomatic to severe forms and mainly involve the respiratory system, in which patients commonly present with fever, cough, and dyspnea [13, 14]. Several studies have reported lymphopenia (absolute lymphocyte counts <1000/mm^3^) as one of the signs of COVID-19 infection; yet, the exact underlying mechanism remains unclear [15–19]. While most patients have a return of lymphocyte counts to normal within the first seven days after admission, some patients may have lymphopenia lasting for 2–4 weeks [15, 16]. While specific treatments for COVID-19 are still under research and development, numerous types of COVID-19 vaccines have been introduced and widely distributed worldwide [20–22]. These vaccines cannot completely prevent infection in vaccinated people; however, they can alter the severity of the infection and associated complications [23–25]. Several skin manifestations have been reported after COVID-19 infection and vaccination, including HZ. A systematic review reported that 3.4% of new-onset skin diseases after COVID-19 infection were cases of HZ [8, 9].

To the best of our knowledge, there remains a gap in our understanding of whether HZ that occurs following COVID-19 infection or vaccination differs in its clinical course from HZ in the general population. This prospective study aimed to compare the demographic data, clinical manifestations, treatments, and outcomes between patients with HZ occurring within 14 days after COVID-19 infection or vaccination and patients with HZ who had no history of COVID-19 infection or vaccination within 14 days.

## 2. Methods

This prospective observational cohort study was conducted at Siriraj Hospital Dermatology Clinic between October 2021 and January 2023. The inclusion criteria were (i) patients diagnosed with HZ by clinical and/or laboratory findings, (ii) age of 18 years or older, and (iii) no previous treatment of this HZ episode. Patients who were lost to follow-up were excluded from the study. Informed consent was obtained from patients who agreed to participate, including the publication of the case details. Demographic data, clinical manifestations, pain scores using a numerical rating scale, and history of COVID-19 infection and COVID-19 vaccination were collected during the first visit. The duration of antiviral treatment, crusting of all lesions (days), and pain scores were recorded at each visit until the patients had no pain and their lesions healed. Disseminated herpes zoster was defined according to the Centers for Disease Control and Prevention's (CDC) definition, which involves the presence of primary lesions in 1–3 dermatomes and the presence of lesions outside the primary or adjacent dermatomes or visceral involvement [26]. Confirmation was achieved through an indirect immunofluorescence assay for VZV using a commercial reagent kit containing VZV monoclonal antibodies (Merck, Ltd.). Postherpetic neuralgia was defined as constant pain, intermittent pain without stimulus, and hyperalgesia lasting for at least three months after the healing of skin lesions of HZ [27]. The protocol was approved by the Siriraj Institutional Review Board (*Si* 799/2021).

### 2.1. Data and Statistical Analysis

Descriptive statistics were used to describe the demographic data. Chi-square and Fisher's exact tests were used to compare the differences in the clinical characteristics between groups. Continuous data with normal and nonnormal distributions were compared using an independent *t*-test and the Mann–Whitney *U* test, respectively. A Kaplan–Meier survival curve was applied to show the differences between the survival curves for crust-off time and duration of neuralgia, which were compared using the log-rank test. A *P* value (*P* < 0.05) was considered statistically significant. All statistical analyses were performed using IBM SPSS Statistics for Windows, Version 28.0 (IBM Corp., Armonk, NY, USA).

## 3. Results

A total of 232 patients with HZ were included in this study. Their median age (IQR) was 62 (46, 70) years, and 59.1% of the patients were women. Only 2 (0.9%) patients had a history of zoster vaccination. The Tzanck smear was positive in 180/185 (97.3%) cases. There were 42 (18%) patients with systemic symptoms, most manifesting as fever (54.8%), followed by eye (38.1%), and ear involvement (0.9%). Only one patient had Ramsay–Hunt syndrome. Systemic thymidine kinase-dependent antiviral agents were prescribed to 229 (98.7%) patients for a median treatment duration (IQR) of 7 days (7, 8). Gabapentin, pregabalin, nortriptyline, and amitriptyline were administered for pain control to 150 (64.7%) and 67 (28.9%) patients, respectively. The median duration (IQR) to crust-off was 13 (9–18) days. Hyperpigmentation was observed in 110 (47.4%) patients, and scars were detected in 24 (10.3%) patients. Postherpetic neuralgia occurred in 19/212 (9%) patients, with a median duration (IQR) of 35 (14, 46) days.

Among 232 patients with HZ, 23 (9.9%) patients developed HZ within 14 days of receiving the COVID-19 vaccination and 4 (1.7%) patients developed HZ within 14 days of being infected with COVID-19. Of the 23 patients with HZ who had recently received vaccination, 12 (52.2%) patients received mRNA vaccines (BNT162b2; Pfizer and mRNA-1273; Moderna) and 11 (47.8%) received the nonreplicating viral vector (ChAdOx1 nCoV-19; AstraZeneca) COVID-19 vaccine as the second to fourth dose. The mean duration (SD) between HZ and vaccination was 5.7 (4.6) days. There have been four cases of HZ following a recent COVID-19 infection. None had symptoms of deoxygenation or pneumonia due to COVID-19, and all patients were treated as outpatients with supportive therapy or the addition of antiviral drugs (i.e., favipiravir or molnupiravir). The median duration (IQR) between HZ and COVID-19 infection was 8.5 (5.3, 13.3) days.

**Figure 1:**
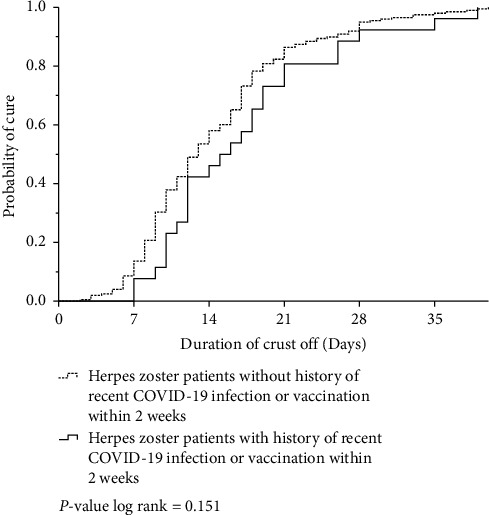
Kaplan–Meier curve of the duration to crust-off of herpes zoster patients with and without a history of recent COVID-19 infection or vaccination within 2 weeks (log rank of the *P* value = 0.151).

**Figure 2:**
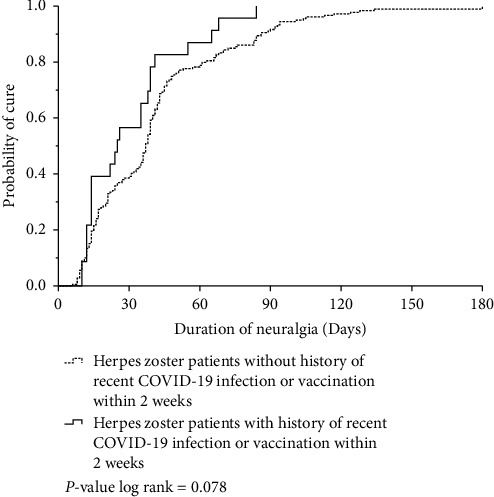
Kaplan–Meier curve of the duration of neuralgia between the onset of herpes zoster and cure in patients with and without a history of recent COVID-19 infection or vaccination within 2 weeks (log rank of the *P* value = 0.078).

The demographic data, clinical characteristics, and treatment outcomes of patients with or without COVID-19 vaccination or infection are shown in Table 1. The proportion of female patients with a history of COVID-19 vaccination or infection was significantly higher than that of those without a history (*P* = 0.035). Patients with a recent COVID-19 infection seemed to have a younger median age (IQR) (42.5 [36.3, 58.5] years) than those in the control group and the recent COVID-19 vaccination group (62 [46.0, 70.5] and 64.0 [58.0, 70.0] years, respectively). The clinical characteristics, systemic symptoms, and postherpetic neuralgia were similar among the three groups of patients. The mean total lymphocyte count (SD) within one month of HZ infection did not differ between patients with and without a history of COVID-19 vaccination or infection (1817.4 (749.2) vs. 1720.9 (784.9), *P* = 0.775). Nine patients were lost to follow-up after their initial visit. The treatment outcomes tended to be worse in patients with HZ and a recent history of COVID-19 infection or vaccination. The median duration to crust-off was prolonged, and there was the higher proportion of scar development; however, this difference was not statistically significant (Table 1 and Figure 1). The duration of neuralgia after the onset of HZ was similar between the two groups, as shown in Figure 2.

The subgroup comparison of demographic data, clinical characteristics, and treatment outcomes between patients with a history of COVID-19 vaccination (*N* = 23) and those with no history of COVID-19 infection or vaccination (*N* = 205) revealed a significantly higher proportion of females in the former group (78.3%) than in the latter group (56.6%, *P* = 0.045). Other parameters, including age, immunological status, pain score at the first visit, clinical presentation, duration to crust-off, healing outcomes, duration of postherpetic neuralgia, and the proportion of cases with postherpetic neuralgia, did not show any statistically significant differences.

Five patients (2.2%) in this study experienced these episodes as second recurrent episodes of HZ. Their mean age (SD) was 62.8 (10) years, and 80% were women. None of the patients had received a herpes zoster vaccine previously. The mean duration (SD) between the first HZ episode and these recurrent episodes was 170.2 (107.9) weeks. Four patients had recurrent HZ within six weeks post-COVID-19 vaccination; two had received mRNA vaccines, and two had received ChAdOx1 nCoV-19 vaccines. The mean duration (SD) from vaccination to the development of HZ was 12.5 (9.7) days. One patient experienced recurrent HZ 21 days after the onset of COVID-19.

Eight (3.4%) patients had disseminated HZ, including five (62.5%) women, and their mean age (SD) was 55.5 (16.8) years. Most patients (87.5%) were immunocompetent. One patient developed disseminated HZ 16 days after COVID-19 onset. Among the other seven patients with a history of COVID-19 vaccination, three (42.9%) patients had disseminated HZ within one month after vaccination. Two of these patients received a nonreplicating viral vector (ChAdOx1 nCoV-19; AstraZeneca) COVID-19 vaccine, and one patient received mRNA (BNT162b2; Pfizer).

## 4. Discussion

This prospective cohort study provides comprehensive comparisons of demographic data, clinical manifestations, treatments, and treatment outcomes among three groups of patients with HZ divided according to the onset of HZ after COVID-19 infection or vaccination within 14 days. Of the 232 patients in this study, 9.9% of HZ patients had a history of recent booster doses of COVID-19 vaccination and 1.7% had a history of recent COVID-19 infection. A significantly higher proportion of patients with HZ, who had a history of recent COVID-19 vaccination or infection, were women compared to those with no history. The median age of patients with recent COVID-19 infections tended to be lower than that of other patients. Prodrome symptoms, lesion morphology, dermatomal involvement, complication rates, duration of antiviral drug use, duration to crust-off, and time to remission of postherpetic neuropathic pain were similar among the three groups. The dissemination and recurrence rates of HZ after COVID-19 vaccination were higher than those in controls.

With regard to HZ after COVID-19 vaccination, this association remains controversial. Some studies found an increased risk of HZ after COVID-19 vaccination [28, 29], and some did not [30–34]. HZ could be reactivated after receiving several types of COVID-19 vaccines, such as inactivated, nonreplicating viral vector, and mRNA vaccine [34–36]. The most commonly reported type includes mRNA vaccines, especially BNT162b2, which could be caused by different proportions of COVID-19 vaccine types, available in different countries. Studies in Hong Kong reported that almost half of the HZ events occurred from inactivated vaccines [35], while studies in Italy reported that more than 90% of HZ cases presented after mRNA vaccines [37]. Similarly, in these studies, a nearly equivalent percentage of patients received mRNA and nonreplicating viral vector vaccines prior to HZ reactivation.

This study demonstrated that among cases with HZ who had a recent history of COVID-19 vaccination or infection, the proportion of females was significantly higher than that of those without a history. Similarly, previous studies have reported that the female sex is an additional risk factor for the development of HZ [1, 38]. Additional studies are needed to understand whether women are more likely to develop HZ after COVID-19 vaccination or infection. The mean age of patients with HZ after COVID-19 vaccination was comparable to that of previous studies [35, 36, 39] (range 58.9–62 years). A previous systematic review reported a higher percentage of cases of autoimmune diseases (13.2%) [39], whereas the current study observed only 4.3% cases. Three studies reported that the mean or median duration after COVID-19 vaccination, regardless of the number of doses, to the onset of HZ ranged from 5.8 to 8 days [36, 39, 40]. This is consistent with this study, which reported a duration of 5.7 days after the second to fourth dose of vaccination.

The association of HZ reactivation and COVID-19 might be caused by the reaction between SARS-CoV-2 virus infection and T-cell immune dysfunction, which contributes to a decrease in VZV-specific CMI and subsequently reactivates HZ [41–43]. When patients are infected with COVID-19, a cytokine storm (releasing a large amount of several proinflammatory cytokines) often occurs [44] and is followed by lymphopenia, functional impairment of CD4+ T cells, and a quantitative decrease in monocytes, eosinophils, and especially, CD3+ and CD8+ T cells [37, 41]. This study showed that the age of HZ patients with a recent history of COVID-19 infection was lower than that of the other two groups, which may be explained by the extreme immune response in the young population compared to the elderly. Furthermore, since this study did not find a significant difference in the total lymphocyte count between individuals with and without a history of COVID-19, this suggests that the problem may be related to functional defects rather than the quantity.

The median time from COVID-19 symptoms to the onset of HZ was approximately one week in both this study and a systematic review by Czech and Nishimura [45]. The current study demonstrated that the clinical manifestations of HZ after COVID-19 infection and vaccination did not seem to be more severe than those in the control group. In addition, the treatment duration and the duration of neuralgia were similar. Oral thymidine kinase-dependent antiviral drugs are still the main specific monotherapy in this study and in previous studies [36, 39, 40, 46]. Regarding the treatment outcomes, HZ patients with a recent history of COVID-19 vaccination or infection tend to have a prolonged median duration to crust-off and scar development; however, this difference was not statistically significant. Close observation and prolonged follow-up may be required in cases with HZ and a history of COVID-19 vaccination or infection.

Disseminated HZ was more likely to be reported in patients with recent COVID-19 vaccinations than in controls; however, this finding was not statistically significant. Several previous studies have reported that HZ was disseminated after COVID-19 vaccination, including inactivated vaccines, nonreplicating viral vectors, and mRNA vaccines, in patients older than 65 years of age or in immunocompromised hosts [47–51]. However, this study reported that only one patient with disseminated HZ after vaccination was immunocompromised, and only 28.6% of patients were older than 65 years of age. Future studies should investigate whether COVID-19 vaccination increases the risk of disseminated HZ. However, physicians should be vigilant about the development of disseminated HZ after COVID-19 vaccination, especially in elderly or immunosuppressed patients.

The rate of recurrence of HZ was reported to be ranging from 5.3 to 6.4% of patients with initial HZ, and the significant risk factors were older age, female sex, and immunosuppressed status [38, 52, 53]. This finding is consistent with the current study, where four of five cases of recurrence were in women. A study with the longest follow-up period of 13 years reported that the mean interval between episodes was 1,062.9 days or 151.8 weeks, which was slightly different from that in this study, in which the interval was 170.2 weeks. Notably, four out of five patients with a history of HZ had been vaccinated or infected with COVID-19 prior to this episode, within a range of 1–21 days. The mean interval between HZ episodes in patients with a recent COVID-19 vaccination was less than that in those without by approximately 10 weeks. This raises the question of whether vaccination or COVID-19 is associated with HZ recurrence.

### 4.1. Limitations

This prospective cohort study was conducted in an outpatient dermatology clinic. Hospitalized patients, such as those with HZ prior to or simultaneously diagnosed with COVID-19, were not included. The association interval between COVID-19 infection or vaccination and HZ has varied in previous studies. This study examined the period within 14 days of COVID-19 vaccination/infection to elucidate the effect of COVID-19 and HZ. Therefore, a small sample size was allocated to the post-COVID-19 group, which may have occurred during hospitalization. Near the end of the study period, booster vaccinations were not encouraged, resulting in a limited sample size of patients with HZ receiving recent vaccinations.

## 5. Conclusions

HZ after COVID-19 vaccination was more common in females compared to those without a history, while HZ after COVID-19 infection tended to occur in younger patients. Most patients developed HZ after COVID-19 vaccination or infection within 5–10 days. Although the clinical manifestations, treatment outcomes, and complications were not significantly different from those of patients with HZ and without recent infection or vaccination, recurrent HZ and HZ dissemination were reported in a higher proportion of patients with HZ after COVID-19 vaccination or infection. Physicians should be aware of the potential for disseminated or recurrent HZ infection following COVID-19 vaccination or infection, especially in elderly or immunocompromised patients.

## Figures and Tables

**Table 1:** Comparison between herpes zoster patients with and without a history of recent COVID-19 infection or vaccination within 2 weeks.

Demographic data	Herpes zoster cases without a recent history of COVID-19 vaccination or infection *N*/total (%) (*N* = 205)	Herpes zoster cases with a recent history of COVID-19 vaccination or infection			*P* value^†^
		Total *N*/total (%) (*N* = 27)	Cases with only a recent history of COVID-19 vaccination *N*/total (%) (*N* = 23)	Cases with only a recent history of COVID-19 infection *N*/total (%) (*N* = 4)	
Sex					
Male	89 (43.4)	6 (22.2)	5 (21.7)	1 (25.0)	0.035^*∗*^
Female	116 (56.6)	21 (77.8)	18 (78.3)	3 (75.0)	
Median age (IQR) (years)	62 (46.0, 70.5)	62 (38.0, 70.0)	64.0 (58.0, 70.0)	42.5 (36.3, 58.5)	0.981
Immunological status					
Immunocompromised	58 (28.3)	5 (18.5)	4 (17.4)	1 (25.0)	0.283
Immunocompetent	147 (71.7)	22 (81.5)	19 (82.6)	3 (75.0)	
Median pain score at first visit (IQR)	5 (2.0, 7.0)	4 (2.0, 6.5)	5.0 ± 3.0	4.0 ± 2.9	0.443
Clinical presentation					
Dissemination	6 (2.9)	2 (7.4)	2 (8.7)	0	0.282
Eye involvement	7 (3.4)	1 (3.7)	1 (4.3)	0	1.000
Median duration to crust-off (IQR) (days)	13 (9.0, 18.0)	15.5 (10.8, 21.0)	14.5 (10.8, 21.0)	19.0 (10.0, 34.0)	0.085
Outcomes of healing (*N* = 223)	*N* = 197	*N* = 26	*N* = 22	*N* = 4	
Normal skin	50 (25.4)	10 (38.5)	10 (45.5)	0	0.157
Nonnormal skin	147 (74.6)	16 (61.5)	12 (54.5)	4 (100.0)	
(i) Hypopigmented lesions	28/147 (19.0)	1/16 (6.3)	1/12 (8.3)	0	0.096
(ii) Hyperpigmented lesions	100/147 (68.0)	10/16 (62.5)	7/12 (58.3)	3/4 (75.0)	
(iii) Scars	19/147 (12.9)	5/16 (31.3)	4/12 (33.3)	1/4 (25.0)	
Median duration of postherpetic neuralgia (IQR) (days)	36.0 (14.5, 46.5)	24.5 (12.0, 39.5)	24.5 (12.0, 39.5)	30.5 (14.5, 42.8)	0.170
Postherpetic neuralgia (pain ≥3 months after onset)	18/187 (9.6)	1/25 (4)	1/21 (4.8)	0	0.707

^*∗*^Statistical *P* value less than 0.05 indicates statistical significance. ^†^The *P* value was derived from statistical analysis comparing herpes zoster cases without a recent history of COVID-19 vaccination or infection (*N* = 205) to herpes zoster cases with a recent history of COVID-19 vaccination or infection (*N* = 27).

## Data Availability

The datasets generated or analyzed in this study are available from the corresponding author upon reasonable request.
